# Accuracy of synthesized right-sided/posterior chest lead electrocardiograms

**DOI:** 10.1186/cc13325

**Published:** 2014-03-17

**Authors:** Y Saitoh, Y Goseki, A Yamashina

**Affiliations:** 1 UniversitairZiekenhuis Brussel, Vrije Universiteit Brussel, Brussels, Belgium; 2 Tokyo Medical University, Tokyo, Japan

## Introduction

Right-sided precordial leads (V3R to V5R) and posterior chest leads (V7 to V9) provide important information for the right ventricle and posterior wall. These additional lead electrocardiograms (ECGs) improve diagnostic value in acute coronary syndrome patients [1]. However, these additional electrocardiograms are not routinely recorded due to the time-consuming procedure involved. Recently these synthesized six additional lead ECGs using the standard 12-lead ECG system (Nihonkoden Co. Ltd) have been developed [2],[3]. But the accuracy is not clear. The purpose of the present study was to evaluate the accuracy of synthesized ECGs at the ST part.

## Methods

Standard 12-lead and actual V3R to V5R, V7 to V9 lead ECGs at Tokyo Medical University Hospital were successfully recorded and compared with synthesized ECGs at the J point, M point that was defined for the point after 1/16 RR interval and T wave amplitude. ECGs of the complete right branch block, complete left branch block and pacing rhythm were excluded.

## Results

**Figure 1:**
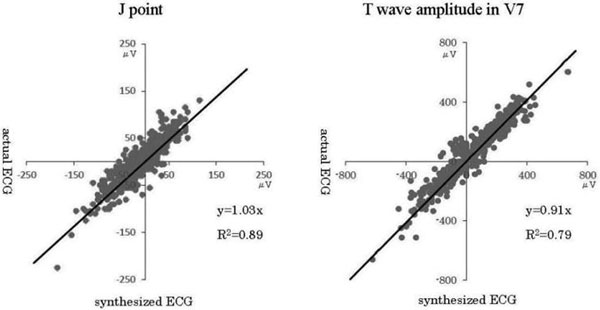
**Correlation between actual and synthesized ECG at V7**.

A total of 1,216 ECGs were correctly recorded. The differences of actual and synthesized at the J point, M point and T wave amplitude were very small. Means of the difference ± 2SD were V3R/V4R/V5R/V7/ V8/V9: J point, 17 ± 1/14 ± 1/13 ± 1/12 ± 1/15 ± 1/18 ± 1 μV; M point, 15 ± 1/13 ± 1/12 ± 1/12 ± 1/12 ± 1/13 ± 1 μV; and T wave amplitude, 20 ± 3/32 ± 2/16 ± 2/37 ± 2/39 ± 2/43 ± 3 μV. There were positive correlations between all actual and synthesized ECGs of J point, M point and T wave amplitude (Figure 1).

## Conclusion

The ST part of synthesized V3R to V5R and V7 to V9 lead ECGs appears to be highly reliable. Synthesized additional lead ECGs might be useful to diagnose ischemic heart disease.
